# Erythritol as sweetener—wherefrom and whereto?

**DOI:** 10.1007/s00253-017-8654-1

**Published:** 2017-12-01

**Authors:** K. Regnat, R. L. Mach, A. R. Mach-Aigner

**Affiliations:** Institute of Chemical, Environmental and Biological Engineering, TU Wien, Gumpendorfer Str. 1a, 1060 Wien, Austria

**Keywords:** Erythritol, Sugar alcohols, Polyols, Sweetener, Sugar, Sugar alternatives

## Abstract

Erythritol is a naturally abundant sweetener gaining more and more importance especially within the food industry. It is widely used as sweetener in calorie-reduced food, candies, or bakery products. In research focusing on sugar alternatives, erythritol is a key issue due to its, compared to other polyols, challenging production. It cannot be chemically synthesized in a commercially worthwhile way resulting in a switch to biotechnological production. In this area, research efforts have been made to improve concentration, productivity, and yield. This mini review will give an overview on the attempts to improve erythritol production as well as their development over time.

## Introduction

Because of today’s lifestyle, the number of people suffering from diabetes mellitus and obesity is increasing. The desire of the customers to regain their health created a whole market of non-sugar and non-caloric or non-nutrient foods. An important part of this market is the production of sugar alcohols, the so-called polyols. The applications vary from food over cosmetics to pharmaceuticals. Whereas polyols like sorbitol, xylitol, mannitol, lactitol, and maltitol are already established and widely used as sugar alternatives for quite a while, erythritol is still developing its whole potential (Billaux et al. 1991; Goossens and Roper 1994). As the production of erythritol is more difficult than of the other polyols, intensive research was performed to optimize its production in terms of improving erythritol concentration, productivity rate, and/or yield. Reports on erythritol reflect the scientific and commercial history of erythritol production. Early studies collected data and information about naturally producing organisms. Later investigations focused on media and cultivation optimization as well as metabolic pathway engineering to increase the amount of produced erythritol. Then, research split into two directions. One focused the discovery of alternative, suitable organisms, like bacteria or filamentous fungi, to open up the range of optimization parameters. The other research direction focused on metabolic pathway engineering or genetic engineering to improve yield and productivity as well as to allow the use of inexpensive and abundant substrates. This review will present the history of erythritol production-related research from a more commercial viewpoint moving towards sustainability and fundamental research.

## Erythritol

**Fig. 1:**
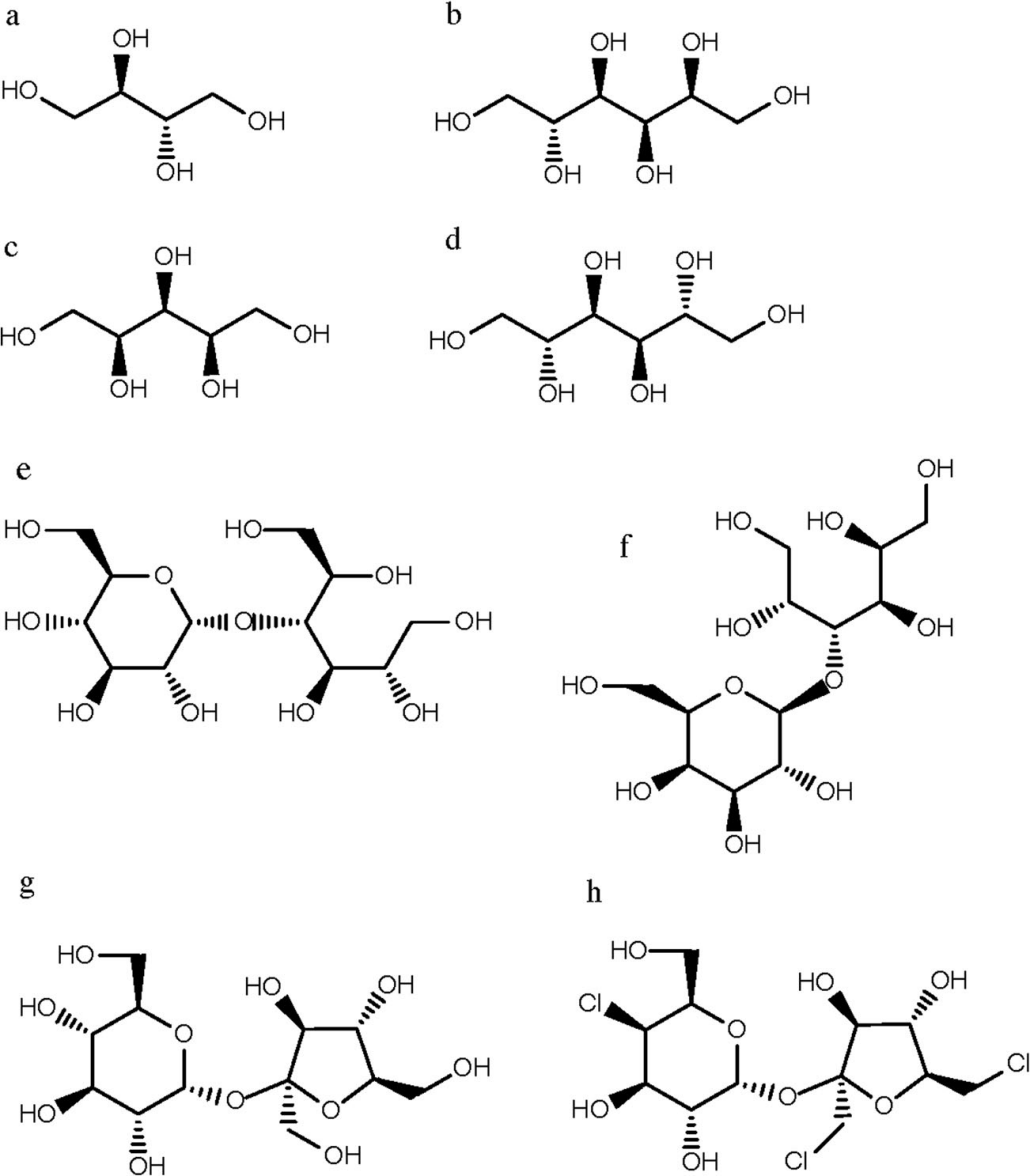
Chemical structures of sweeteners discussed in this review, namely **a** erythritol, **b** sorbitol, **c** xylitol, **d** mannitol, **e** maltitol, **f** lactitol, **g** sucrose, and **h** sucralose

Erythritol ((2R,3S)-Butan-1,2,3,4-tetrol) belongs to the family of sugar alcohols also known as polyols, which are formed due to hydrolysation processes of the aldehyde or ketone group in various carbohydrates (Billaux et al. 1991). The chemical structure of erythritol and the other sweeteners discussed in this review are provided in Fig. 1. Polyols are naturally abundant in fruits and vegetables, like grapes and mushrooms as well as in fermented foods like soy sauce (Bernt et al. 1996; Shindou et al. 1988; Yoshida et al. 1986). The most valuable properties of these sugar alcohols are their sweetness and low calorie content combined with being non-cariogenic (Mäkinen 1994). For an overview on these properties, see Table 1. Within the sugar alcohols, erythritol plays a somehow extraordinary part. It consists of only four carbon atoms and has therefore the smallest molecular weight of all sugar alcohols, which is associated with slightly different physical and chemical properties. Erythritol is also a symmetrical molecule and therefore exists only in one form, the meso-form (Fig. 1). It forms anhydrous crystals with a moderate sweetness of 60–80% of sucrose (Goossens and Gonze 1996) (Table 1). However, as an advantage, it can be mixed with more intense sugars due to the absence of any aftertaste (Barbieri et al. 2014; Bernt et al. 1996; Moon et al. 2010). But due to the high production costs of erythritol compared to more intense sweeteners, it is not primarily chosen for its sweetness synergy. As a more important feature, erythritol can improve the mouth feeling and can mask certain unwanted aftertastes such as astringency and the irritant effect of intense sweeteners (de Cock 2012). When dissolved, erythritol exhibits a strong cooling effect due to its high negative heat of solution (Park et al. 2005). Along with the artificial sweetener sucralose, it is the only polyol that is non-caloric, providing no energy to the body. The majority of erythritol cannot be metabolized by the human body and is excreted unmodified into the urine without changing blood glucose and insulin levels (de Cock 2012; Efsa Panel on Dietetic Products and Allergies 2011; Grabitske and Slavin 2008). The latter is a stand-alone property of erythritol among the commonly used polyols and allows its usage as sweetener in specialized food for diabetics or people suffering obesity (Wheeler and Pi-Sunyer 2008). It also means that a severe disadvantage of other polyols, namely sorbitol and xylitol, leading to diarrhea is eliminated (Bernt et al. 1996; de Cock 2012). Only a little amount, less than 10%, undergoes a reversible metabolic reaction like the dehydration to d- or l-erythrulose (Moon et al. 2010; Park et al. 2005, 2016). Finally, erythritol is also a free radical scavenger with the ability to potentially exercise its anti-oxidant activity while circulating the body before it is excreted into the urine (de Cock 2012; den Hartog et al. 2010). For an overview on the biological effectiveness and reported side effects of the sweeteners discussed in this review, see Table 2.

**Table 1:** Properties of sweeteners discussed in this review

Sweetener	Systematic name	Synonyms	Glycemic index^1^	Caloric value (kcal/g)^1^	Sweetness^2^
Sucrose	(2*R*,3*R*,4*S*,5*S*,6*R*)-2-[(2*S*,3*S*,4*S*,5*R*)-3,4-dihydroxy-2,5-bis(hydroxymethyl)oxolan-2-yl]oxy-6-(hydroxymethyl)oxane-3,4,5-triol	Sugar Saccharose	65.0^3^	3.9	1.0
Erythritol	(2R,3S)-Butane-1,2,3,4-tetrol	Erythrit Meso-erythritol Tetrahydroxybutane	0.0	0.2	0.6–0.8
Xylitol	D-erythro-pentitol	Xylit Birkenzucker	13.0	2.4	1.0
Mannitol	D-Mannitol	Mannite	0.0	1.6	0.5–0.7
Sorbitol	D-Glucitol	D-Glucitol syrup Sorbit Sorbol	9.0	2.7	0.5–0.7
Maltitol	4-O-α-d-Glucopyranosyl-d-glucitol	Dried maltitol syrup Hydrogenated maltose Maltitol syrup powder	35.0	2.1	0.9
Lactitol	4-O-β-l-Galactopyranosyl-l-glucitol	Lactit Lactobiosit Lactositol	6.0	1.9	0.3–0.4
Sucralose	(1 → 6)-Dichloro-(1 → 6)-dideoxy-β-D-fructofuranosyl-4-chloro-4-deoxy-α-D-galactopyranoside	Trichlorosucrose TGS Splenda	0.0	0.0	320.0–1000

^1^According to (Livesey 2003)

^2^According to (Canada 2016)

^3^With glucose = 100

**Table 2:** Biological effectiveness and reported side effects of polyols (Grembecka 2015) and sucralose discussed in this review

Sweetener	Biological effectiveness	Reported side effects
Erythritol	Non-caloric Non-glycemic Non-cariogenic High digestive tolerance Free radical scavenger Non-acidogenicity Anti-oxidative and endothelium-protective properties Increases malabsorption of fructose	Non-observed Symptoms of overconsumption are flatulence and laxation
Xylitol	Low calorie index Low glycemic index Non-cariogenic, improves dental health Increases saliva production, which helps in treating xerostomia Protects salivary proteins, has a protein-stabilizing effect Improves breath odor Reduces infections in the mouth and nasopharynx Anti-ketogenic—decreases serum-free fatty acid levels and improves peripheral glucose utilization Favors absorption of calcium and B vitamins Inhibits growth of yeast, including *Candida albicans* Decreases glycation of proteins, reduces AGEs Helps to maintain healthy gut function	Temporary laxation and gastrointestinal discomfort
Mannitol	Low calorie index Reduces the rise in blood glucose and insulin levels Non-cariogenic When inhaled, helpful in mucus and cough clearance in asthmatics and other hypersecretory diseases	In amounts greater than 20 mg/kg body weight may cause abdominal pain, excessive gas (flatulence), loose stools or diarrhea
Sorbitol	Reduced calorie value Low glycemic index Non-cariogenic	Osmotic diarrhea as a result of intestinal malabsorption when ingested dose is greater than 50 g per day Consumption of 20–30 g/day results in abdominal pain
Maltitol	Reduced calorie value Low glycemic index Non-cariogenic Increases mineral bioavailability in human and rats Combination with short-chain fructo-oligosaccharides in sugar-free food product formulations results in lower postprandial glycemic responses	Abdominal bloating and laxative effect when consumed in large quantities
Lactitol	Reduced calorie value Non-cariogenic Increases the growth of probiotic bacteria Reduces the population of putrefactive bacteria Lowers the intestinal pH Role in treating encephalopathy and constipation Increases mineral bioavailability in human and rats	Bloating and flatulence after an intake more than 20 g in a single dose
Sucralose	Non-caloric Non-glycemic Non-carcinogenic Poorly absorbed and no dechlorination Not accumulated in fat; readily eliminated^1^	Not recommended for fructose-intolerant persons Induces glucose-intolerance by altering gut microbiota^2^

^1^According to (Magnuson et al. 2017)

^2^According to (Suez et al. 2014)

## Applications

Although it was firstly isolated in 1852, it took until 1990 for erythritol to become present on the Japanese market as a new natural sweetener (Boesten et al. 2015). The range of applications for erythritol is still growing. It can currently be found on its own, or in combination with other polyols in foods, cosmetics, and pharmaceuticals.

To date, the use of erythritol in foods has been approved in more than 60 countries, including Europe, the USA, Japan, Canada, Mexico, Brazil, Argentina, Turkey, Russia, China, India, Australia and New Zealand (Boesten et al. 2015; de Cock 2012). Within the food sector, erythritol is mainly utilized as sweetener to balance the finished product with regard to its sensory characteristics, such as flavor, color, and texture. Erythritol can therefore be used to produce no-sugar added, reduced-sugar, or sugar-free alternatives. Erythritol as sugar replacement can be found as tabletop sweetener, in beverages, chewing gum, chocolate, candies, and in bakery products (de Cock 2012). Due to its mild sweetness, it allows a volume-for-volume replacement of sugar, whereas for example, sucralose that has a much higher sweetness needs fillers and even then has a noticeably different texture in baked products. With regard to sucralose, it also needs to be considered that it is a chemically synthesized substance that does not naturally occur in nature. As a consequence, it currently accumulates in the environment due to the lack of sufficient natural degradation mechanisms (Lubick 2008).

Polyols are commonly used within the personal care industry like the cosmetic or toiletries sector. They are more and more incorporated as excipients in the manufacture of care products like toothpaste, mouthwashes, creams and lotions, make-up, perfumes, or deodorants. Due to its humectant function as well as its pleasant taste, its sweetness and its non-cariogenic properties, erythritol can be used as base for toothpaste and mouthwash recipes (EPA European association of polyol producers 2017). It gives toothpastes the required viscosity and humectancy. Additionally, erythritol inhibits the growth of *Streptococcus mutans* and acts as caries limiting in combination with xylitol (de Cock et al. 2016; Grembecka 2015). Further, it was found that a 3-year consumption of erythritol-containing candies by 7- to 8-year-old children resulted in reduced plaque growth, lower levels of plaque acetic acid, and propionic acid (Grembecka 2015; Runnel et al. 2013).

Erythritol can be used in a wide range of solid and liquid formulations, including granulated powders, tablets, tablet coating, consumer-friendly lozenges, medicated chewing gum, syrups, and as mentioned before, as oral care products (Michaud and Haest 2003). For pharmaceutical use, its interaction with water and its high stability in temperature and in acid or alkaline environments is a key (Grembecka 2015). Because of its properties, erythritol as excipient offers good flowability and stability, making it an ideal carrier for actives in sachets and capsules. More and more active ingredients are derived from biotechnological processes, which are often very efficient but also extremely reactive. Using the non-reducing sugar erythritol instead of lactose, which is the most commonly used pharmaceutical excipient, the unwanted reaction between the amino groups of the active and the reducing sugar can be prevented. Therefore, lactose is more often being replaced by erythritol. Besides this, non hygroscopic polyols need to be used when a very water-sensitive active has to be reformulated (EPA European association of polyol producers 2017).

## Production—history and development

To extract erythritol from its natural sources, like fruits or vegetables, is not practical because of their low erythritol contents. And in contrast to the other polyols, erythritol is not favored being produced via chemical synthesis. The needed high temperatures as well as the nickel catalyst result in a cost-ineffective reaction with a low product yield (Park et al. 2005; Pfeifer et al. 1960). When in 1950, traces of erythritol were found in the residue of Cuban blackstrap molasses fermented by yeast, a flourishing new possibility opened up: the biotechnological production of erythritol (Deng et al. 2012). Please see Table 3 for an overview on the process parameters of the following discussed strategies or microorganisms.

**Table 3:** Process parameters for erythritol production of the discussed genetically modified microorganisms

Microorganism	Genetic modification	Substrate	Concentration (g/l)	Yield (g/g)	Productivity (g/l/h)	Reference
Yeast						
*T. megachiliensis* SN-G42	Mutagenesis	Glucose	164.8	nd^1^	2	(Ishizuka et al. 1989), (Sawada et al. 2009)
*C. magnoliae* JH110	Mutagenesis	Glucose	200	0.43	1.2	(Kohl et al. 2003)
*C. magnoliae* 12-2	Mutagenesis	Glucose	20.3	nd	nd	(Ghezelbash et al. 2014)
*T. oedocephalis* HOG1	Δ*hog1*	Glucose	56.8	0.28	nd	(Li et al. 2016)
*Y. lipolytica* MK1U- pADUTSuc2	*suc2*	Sucrose	52	0.26	0.9	(Mirończuk et al. 2015)
*Y. lipolytica* AMM pADUTSuc2 92h	*suc2*	Sucrose	73	0.37	1.0	(Mirończuk et al. 2015)
*Y. lipolytica* AMM pADUTSuc2 144h	*suc2*	Sucrose	113.9	0.57	0.8	(Mirończuk et al. 2015)
*Y. lipolytica* AJD pADUTSuc2	*suc2*	Sucrose	59.5	0.32	0.9	(Mirończuk et al. 2015)
*Y. lipolytica* AIB pAD-UTGUT1	*gut1*	Sucrose and glycerol	82.2	0.55	0.87	(Rakicka et al. 2017)
*Y. lipolytica* AMM pAD-GND1	*gnd1*	Glycerol	40.2	0.4	0.43	(Mirończuk et al. 2017)
*Y. lipolytica* AMM pAD-ZWF1	*zwf1*	Glycerol	42.5	0.43	0.45	(Mirończuk et al. 2017)
*Y. lipolytica* AMM pAD-TLK1	*tkl1*	Glycerol	51.1	0.51	0.54	(Mirończuk et al. 2017)
*Y. lipolytica* ΔYALI0F01606g	*eyk1*	Glycerol	nd	0.49	0.59	(Carly et al. 2017)
Bacteria						
*O. oeni*		Glucose	nd	nd	nd	(Ortiz et al. 2013)
*L. sanfranciscencis*		Glucose	nd	nd	nd	(Ortiz et al. 2013)
*Synechocystis* PCC6803	*err1/e4pp*	None^2^	nd	nd	nd	(van der Woude et al. 2016)
Filamentous fungi						
*T. reesei*	*err1*	Wheat straw	nd	nd	nd	(Jovanovic et al. 2013)

^1^nd means not determined

^2^The strain is cultivated under moderate intensity white-light illumination or high intensity illumination for production via photosynthesis

### Production in yeast

The production of erythritol was first observed in yeasts and yeast-like fungi. A strain probably belonging to the genus *Torula* was able to convert 35–40% of utilized glucose into erythritol. However, at the very beginning, it became clear, that although the production of erythritol is inherent in yeast, the fermentation conditions influence the yield in a large scale (Hajny et al. 1964). To understand the connection between metabolism and the fermentation parameters used, it is important to have a look at the natural production of erythritol in yeast. Erythritol is produced via the pentose phosphate pathway, in which d-erythrose-4-phosphate is dephosphorylated to d-erythrose and then reduced to erythritol. This pathway serves various purposes: the production and provision of reduction power in form of NADPH for cellular reactions, the production of precursors for the nucleotide and amino acid biosynthesis, and on its own as compatible solute to protect and stabilize enzymes facilitating cellular functions under osmotic conditions (Brown 1978; Moon et al. 2010). Its function as osmo-protectant is the reason for using osmophilic yeasts such as *Moniliella pollinis*, *Trichosporonoides megachiliensis*, *Aureobasidium* sp*.*, *Trigonopsis variabilis*, *Trichosporon* sp*.*, *Torula* sp*.*, and *Candida magnoliae* as production strains (Chattopadhyay et al. 2014; Grembecka 2015; Lin et al. 2010). They are mainly producing erythritol when encountering salt or osmotic stress (Yang et al. 2015). However, unfavorable fermentation conditions can lead to the production of glycerol at the expense of erythritol formation because glycerol is the main osmolyte in yeasts. Consequently, the next important task was finding methods to increase the erythritol yield and minimize the reaction shift towards glycerol production. Two strategies, namely random mutagenesis and fermentation optimization, were implemented. Random mutagenesis by UV and chemical mutagens revealed mutants producing more erythritol and less by-products than their parental strains due to improved activities and expression levels of key enzymes involved in the pentose phosphate pathway (Park et al. 2016). Several high-yield mutation strains such as *T. megachiliensis* SNG-42, *C. magnoliae* JH110, or *C. magnoliae* 12-2 obtained by ultraviolet and chemical mutagenesis are currently used for erythritol production (Li et al. 2016). Besides random mutagenesis, research moved further to targeted gene editing as more and more knowledge about the regulation of erythritol could be obtained. The HOG1 gene encodes a mitogen-activated protein kinase (MAK) that plays an important role in osmo-adaptation and therefore might influence the production of glycerol and erythritol. The created *Trichosporonoides oedocephalis HOG1* knockout mutant produced 1.4-fold more erythritol than its parental strain. Also, the glycerol production was decreased by 71.23% (Li et al. 2016).

Although erythritol-producing strains are able to convert glucose or fructose into erythritol, much higher productivities and yields were achieved by regulating the initial glucose concentration using a fed-batch fermentation with optimized media and vitamin or mineral supplementation (Moon et al. 2010; Park et al. 2016). Optimizing the fermentation parameters, such as controlling the dissolved oxygen and stirring during the process, could reduce the undesired accumulation of glycerol (Li et al. 2016). Additionally, the role of osmotic and salt stress became more important to improve erythritol yields. It was found that a DNA sequence corresponding to the putative response stress element within the erythrose reductase gene from *C. magnolia* is upregulated under osmotic and salt stress conditions. This stress can be caused by high concentrations of sugar, potassium chloride, or sodium chloride in the fermentation broth (Park et al. 2011).

Side by side, while the erythritol production methods were getting more polished and perfected, the search for other carbon sources than glucose or fructose as substrate began. The yeast *Yarrowia lipolytica* can convert pure or crude glycerol into different substances including polyols (Dobrowolski et al. 2016). Glycerol is a renewable feedstock and the main by-product of the bio-diesel production (Mirończuk et al. 2015; Moon et al. 2010). Hence, recombinant strains of *Y. lipolytica* that express the *SUC2* gene from *Saccharomyces cerevisiae* were generated. By expression of this gene, the organism gains the ability to utilize sucrose, which is the main content of molasses, an agro-industrial by-product (Mirończuk et al. 2015). Such approaches are exemplarily reflecting the trend of using inexpensive raw materials for the conversion into value-added products. In addition to inserting the *SUC2* gene, metabolic engineering by overexpressing the *GUT1* gene leads to sucrose and glycerol utilization with efficient erythritol production. The *GUT1* gene functions as glycerol kinase in the phosphorylative glycerol catabolic pathway in yeast. The enzyme is important for its ability to assimilate glycerol that somehow interferes with erythritol formation and complicates downstream processing (Rakicka et al. 2017). Even though, the strain has been genetically modified to produce more erythritol, the metabolic pathway in *Y. lipolytica* has never been described before. Now, a new study showed the formation of erythritol via pentose phosphate pathway trying additionally to identify the key genes involved (Mirończuk et al. 2017). The overexpression of genes involved in the pentose phosphate pathway, transketolase *TKL1*, and two dehydrogenases *ZWF1* and *GND1* revealed a twofold higher erythritol synthesis for the transketolase overexpression strain, showing the importance of this gene for erythritol biosynthesis (Mirończuk et al. 2017). Quite recently, another gene *EYK1*, coding for an erythrulose kinase, was found to enhance erythritol production (Carly et al. 2017).

### Production in bacteria

Not only the desire for more cost-friendly substrates but also the possibility to use other production host organisms than yeasts is growing. The heterofermentative lactic acid bacteria *Oenococcus oeni* produces erythritol in an alternative pathway for NADPH reoxidation during anaerobic glucose metabolism (Veiga-da-Cunha et al. 1993). The formation of erythritol differs from the synthesis in yeasts: glucose-6-phosphate is converted into fructose-6-phoshate. After cleavage into erythrose-4-phosphate and acetyl-phosphate, the erythrose-4-phosphate is first reduced to erythritol-4-phosphate and then dephosphorylated to erythritol (Ortiz et al. 2013; Veiga-da-Cunha et al. 1993). *Lactobacillus sanfranciscencis*, another LAB strain isolated from sourdough, produces erythritol under stress conditions as an additional metabolic product (Ortiz et al. 2013). An interesting aspect of using functional lactic starter cultures with the ability to produce polyols may be the production of novel fermented food naturally sweetened with these low-calorie sugars.

A totally different approach to improve sustainability in erythritol production was performed using a genetic engineered cyanobacteria strain: *Synechocystis* PCC6803. Several characterized erythrose reductases and erythrose-4-phosphatases from fungi were introduced into the genome of *Synechocystis* PCC6803. The expressed enzymes can then convert erythrose-4-phosphate into erythritol. Erythrose-4-phosphate is inherent and is built as a key intermediate of the CO_2_-fixing Calvin Benson Bassham cycle (van der Woude et al. 2016).

### Production in filamentous fungi

Erythrose reductase is the key enzyme for the production of erythritol and a lot of research has been done on the characterization and purification of various reductases in yeasts (Deng et al. 2012). In *Trichoderma reesei*, a filamentous fungus industrially used due to its ability to secrete vast amounts of cellulases and hemicellulases, the erythrose reductase is also naturally present and has already been characterized (Jovanovic et al. 2013). The erythritol production is, like in yeast, part of the pentose phosphate pathway. d-Erythrose-4-phosphate is first dephosphorylated and then further reduced to erythritol. This reaction is NADPH-dependent and reversible (Jovanovic et al. 2013). Although the production of erythritol in this strain is naturally not as high as in yeasts, the organism has other advantages. It is capable of growing on lignocellulosic material (Acebal et al. 1986) and therefore can utilize cheap biowaste materials as sole carbon source. This can be used for the production of erythritol. The strain can perfectly grow and produce erythritol on wheat straw. It was reported that an erythrose reductase overexpression strain leads to a clearly higher erythritol formation on pretreated wheat straw (Jovanovic et al. 2014). With further substrate optimization, strain improvement, pathway engineering, and fermentation optimization, this might be a promising way to bypass the utilization of expensive fermentation substrates.

## Conclusion

Over time, different approaches have been applied to increase the production of erythritol. Because the demand of erythritol increased in a short time, the commercial availability became most important. First priority was a fast optimization of the fermentation parameters for the cultivation of known erythritol-producing organisms and random mutagenesis. Although the enzymes were soon known, and in some cases have already been characterized, just little research focused on the regulation of the expression of the corresponding genes. Only in the last few years, hypotheses about gene regulation started to be enlightened by targeted gene editing. The knowledge achieved from this research turned out to be useful for targeted genetic strain improvements and opened up new parameters for further fermentation optimization. Later on, the question about ecological sustainability arose. Is it justifiable to produce a sugar substitute using a sugar as feedstock, thereby creating a product that is furthermore expensive? The research work performed on the industrial cellulase and hemicellulase production strain *T. reesei* tried to combine all different optimization approaches. The organism is able to degrade lignocellulosic material and can therefore utilize renewable and cheap material, like wheat straw as starting material. The substrate can be pretreated to facilitate analysis and speeding up the degradation by *T. reesei*. The key enzyme for the synthesis of erythritol is naturally present in *T. reesei* and has been successfully expressed and characterized in *Escherichia coli*. Finally, leads an overexpression of this gene to an increase in erythritol synthesis. The ongoing research focuses on getting a whole picture about the pathway of erythritol synthesis, the enzymes involved, and the influence of certain production parameters like osmotic pressure with the aim to gain a value-added product from cheap renewable biomaterial.
